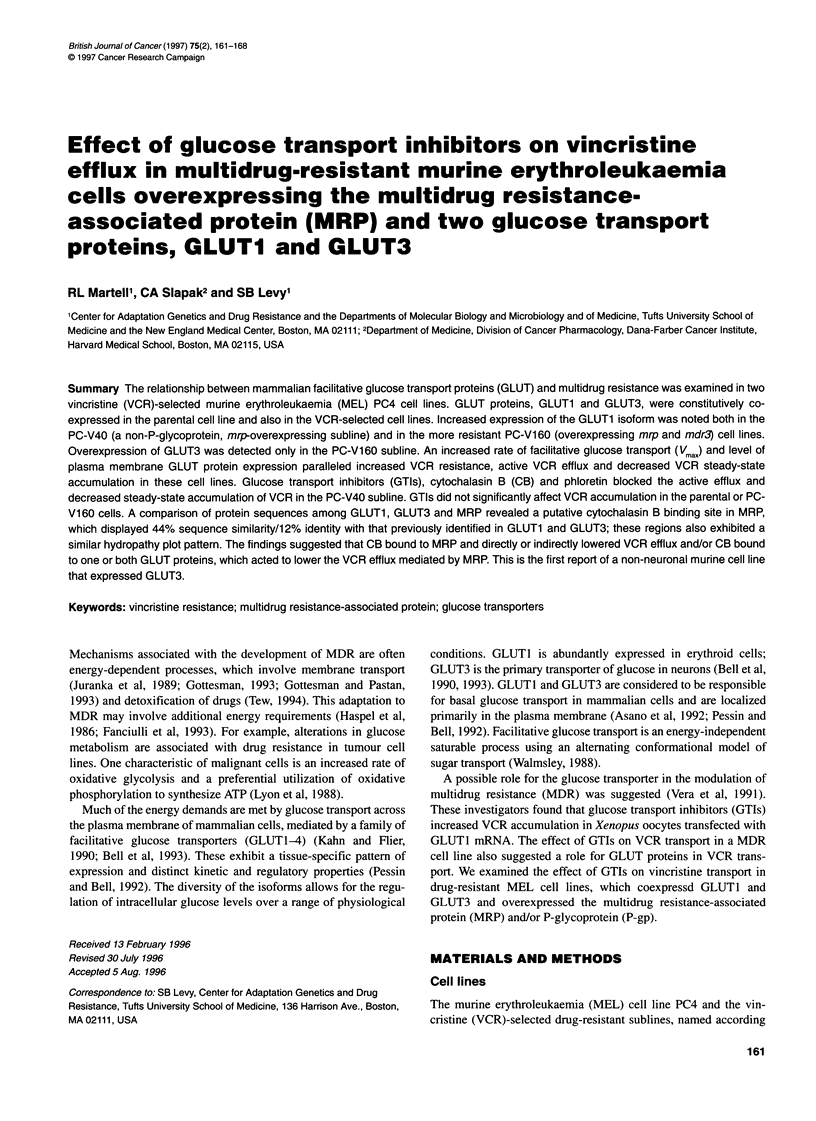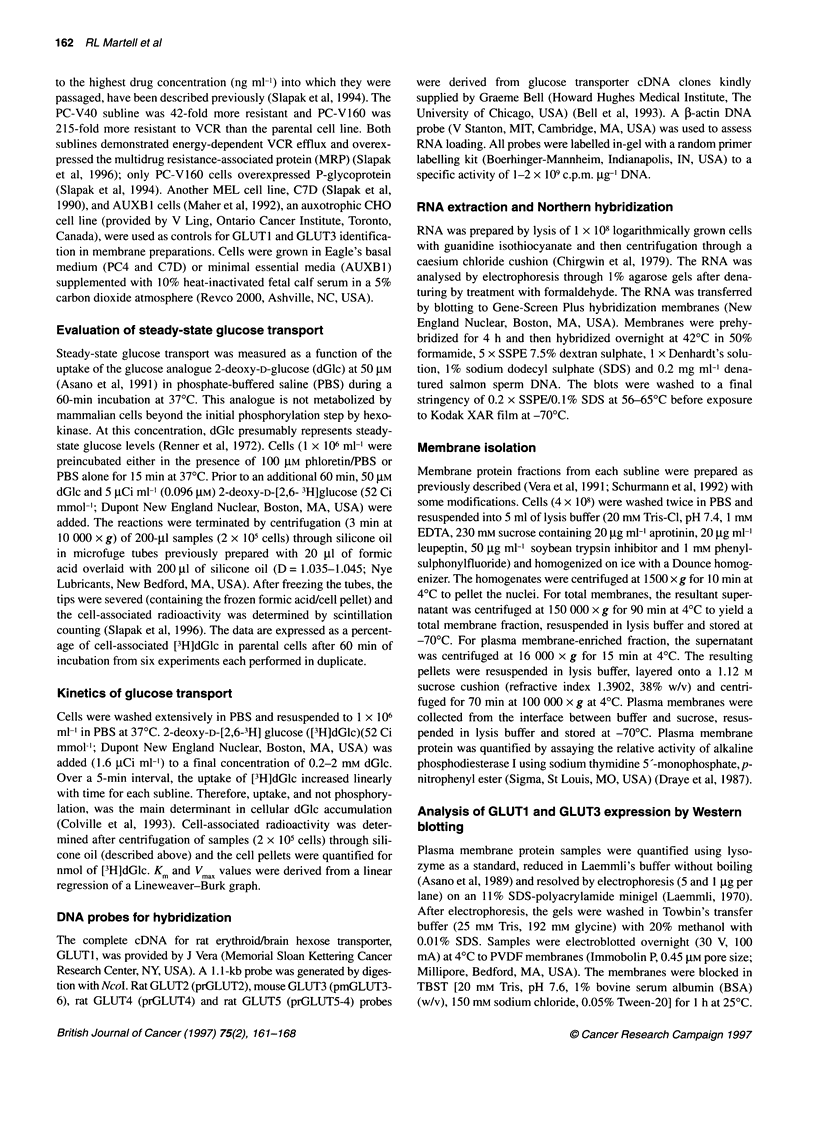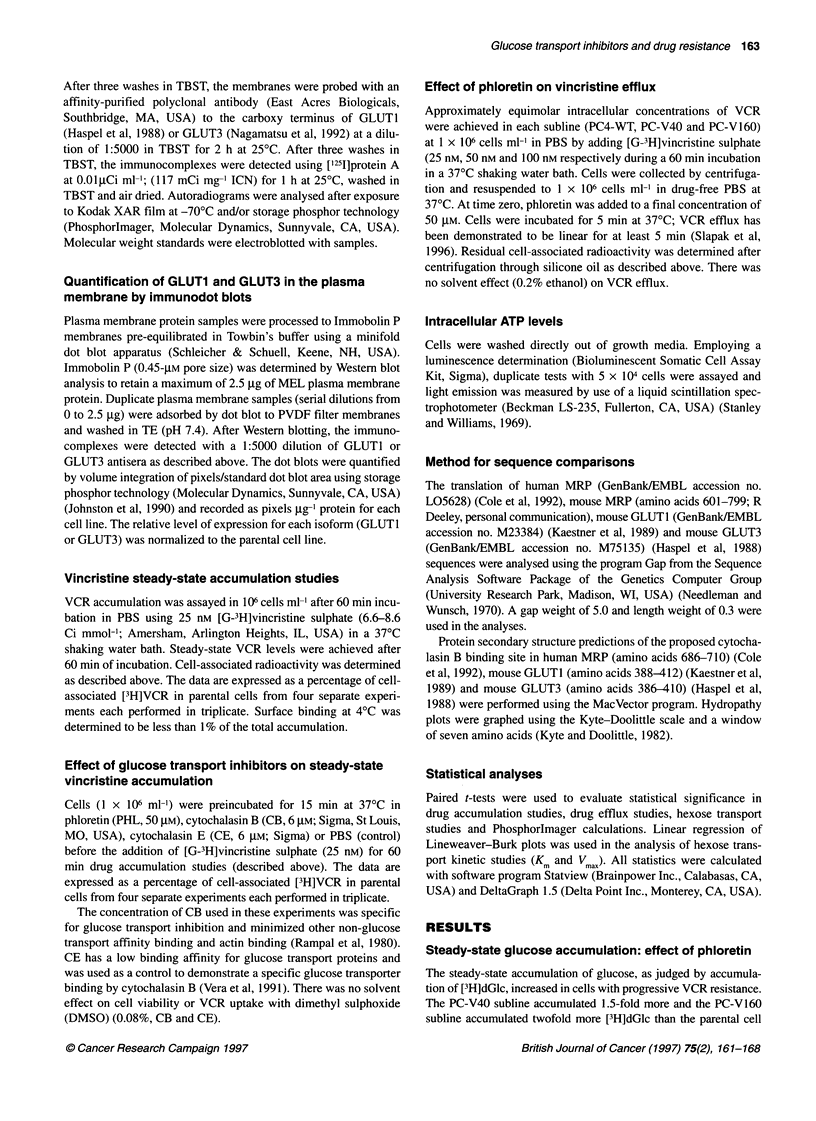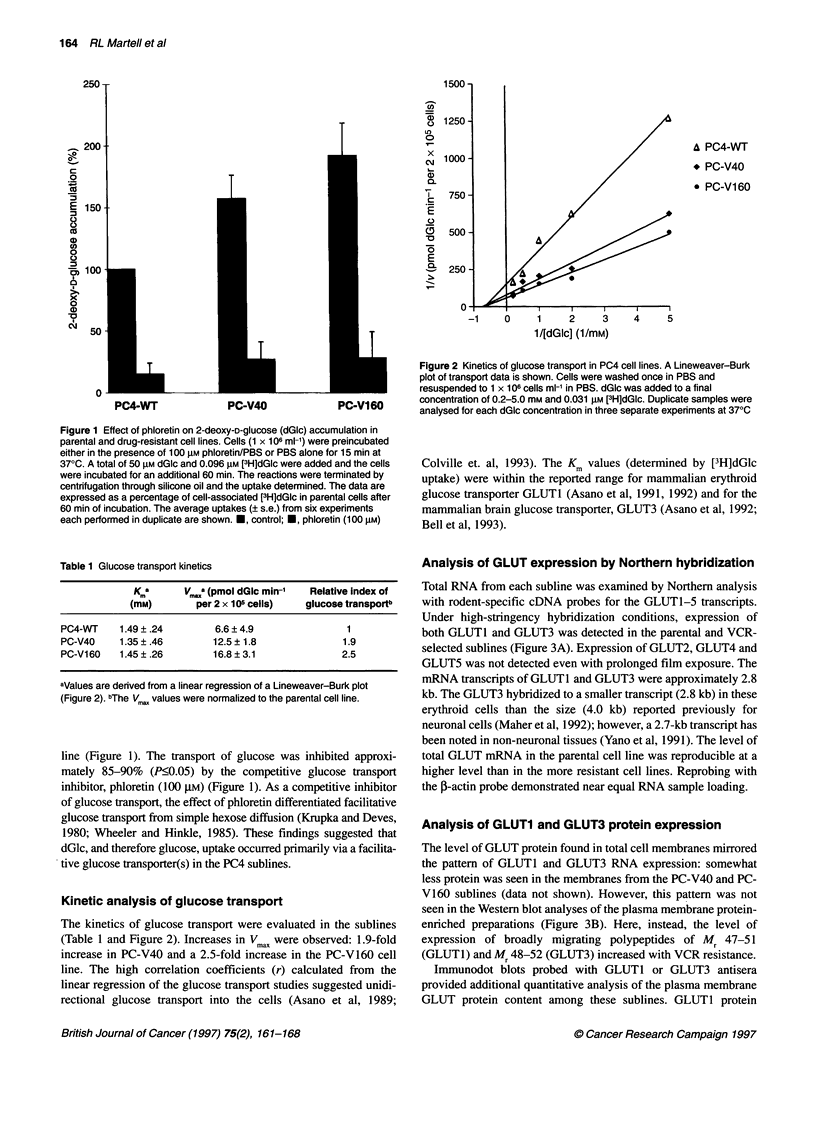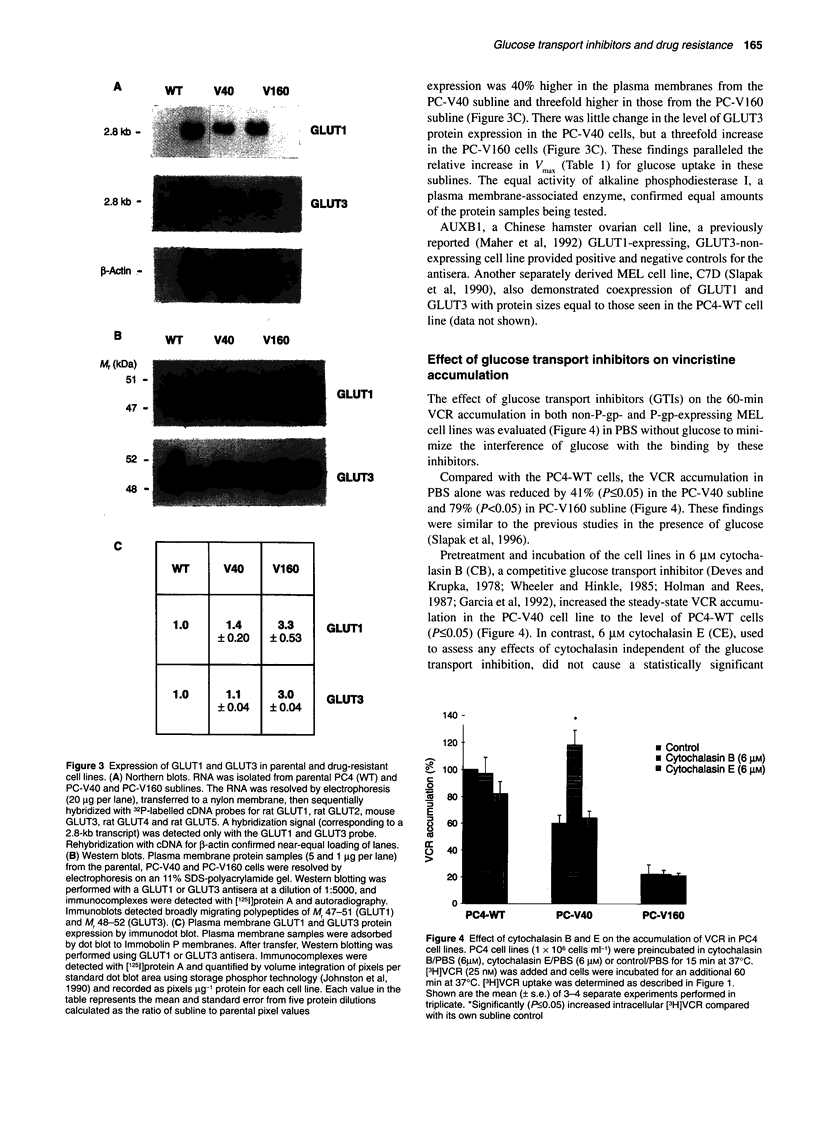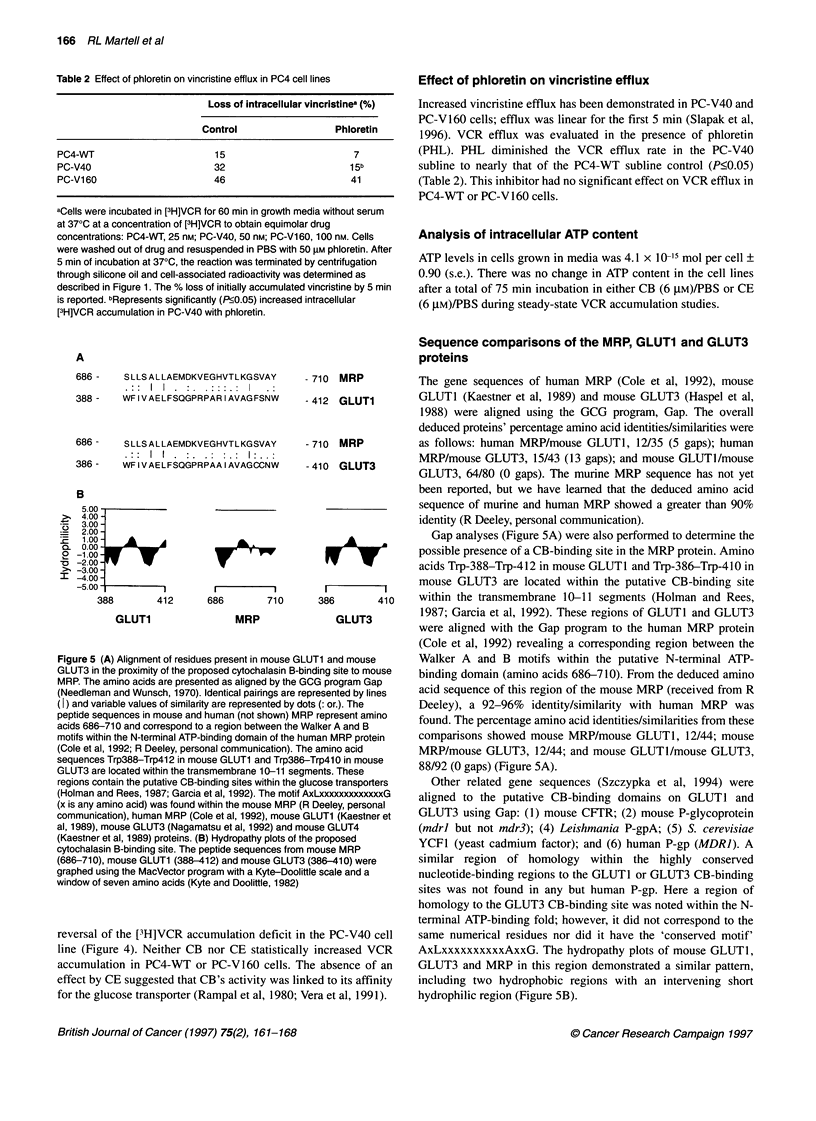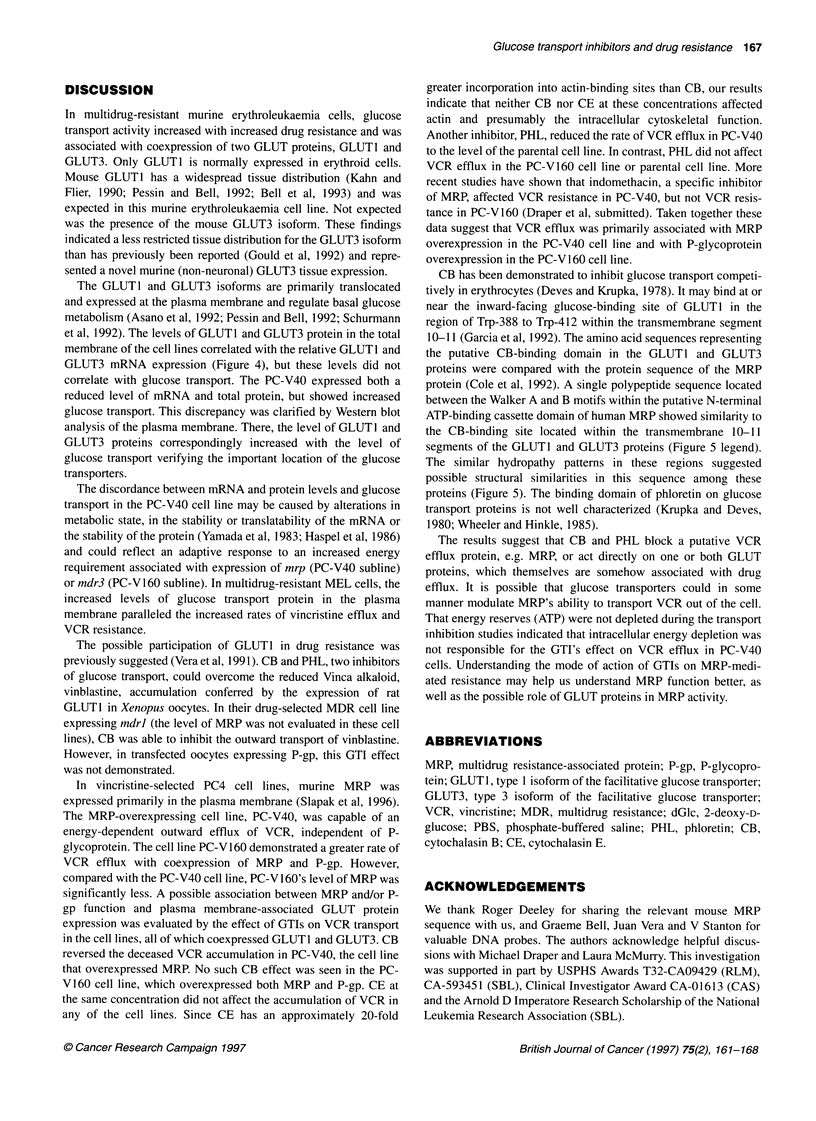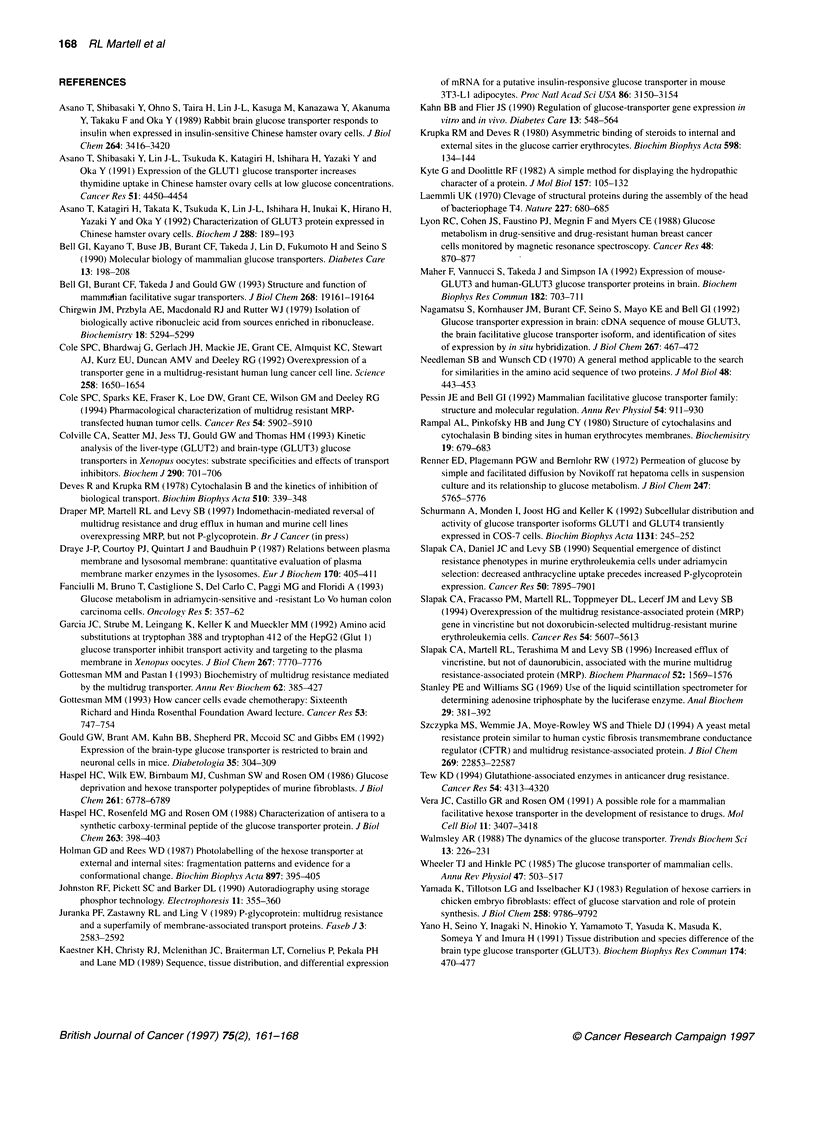# Effect of glucose transport inhibitors on vincristine efflux in multidrug-resistant murine erythroleukaemia cells overexpressing the multidrug resistance-associated protein (MRP) and two glucose transport proteins, GLUT1 and GLUT3.

**DOI:** 10.1038/bjc.1997.27

**Published:** 1997

**Authors:** R. L. Martell, C. A. Slapak, S. B. Levy

**Affiliations:** Center for Adaptation Genetics and Drug Resistance, Tufts University School of Medicine, Boston, MA 02111, USA.

## Abstract

**Images:**


British Journal of Cancer (1997) 75(2), 161–168
© 1997 Cancer Research Campaign

### Effect of glucose transport inhibitors on vincristine efflux in multidrug-resistant murine erythroleukaemia cells overexpressing the multidrug resistance-associated protein (MRP) and two glucose transport proteins, GLUT1 and GLUT3

**RL Martell[1], CA Slapak[2] and SB Levy[1]**

[1]Center for Adaptation Genetics and Drug Resistance and the Departments of Molecular Biology and Microbiology and of Medicine, Tufts University School of Medicine and the New England Medical Center, Boston, MA 02111; [2]Department of Medicine, Division of Cancer Pharmacology, Dana-Farber Cancer Institute, Harvard Medical School, Boston, MA 02115, USA

**Summary** The relationship between mammalian facilitative glucose transport proteins (GLUT) and multidrug resistance was examined in two vincristine (VCR)-selected murine erythroleukaemia (MEL) PC4 cell lines. GLUT proteins, GLUT1 and GLUT3, were constitutively co-expressed in the parental cell line and also in the VCR-selected cell lines. Increased expression of the GLUT1 isoform was noted both in the PC-V40 (a non-P-glycoprotein, *mrp*-overexpressing subline) and in the more resistant PC-V160 (overexpressing *mrp* and *mdr3*) cell lines. Overexpression of GLUT3 was detected only in the PC-V160 subline. An increased rate of facilitative glucose transport ($V_{max}$) and level of plasma membrane GLUT protein expression paralleled increased VCR resistance, active VCR efflux and decreased VCR steady-state accumulation in these cell lines. Glucose transport inhibitors (GTIs), cytochalasin B (CB) and phloretin blocked the active efflux and decreased steady-state accumulation of VCR in the PC-V40 subline. GTIs did not significantly affect VCR accumulation in the parental or PC-V160 cells. A comparison of protein sequences among GLUT1, GLUT3 and MRP revealed a putative cytochalasin B binding site in MRP, which displayed 44% sequence similarity/12% identity with that previously identified in GLUT1 and GLUT3; these regions also exhibited a similar hydropathy plot pattern. The findings suggested that CB bound to MRP and directly or indirectly lowered VCR efflux and/or CB bound to one or both GLUT proteins, which acted to lower the VCR efflux mediated by MRP. This is the first report of a non-neuronal murine cell line that expressed GLUT3.

**Keywords:** vincristine resistance; multidrug resistance-associated protein; glucose transporters

Mechanisms associated with the development of MDR are often energy-dependent processes, which involve membrane transport (Juranka et al, 1989; Gottesman, 1993; Gottesman and Pastan, 1993) and detoxification of drugs (Tew, 1994). This adaptation to MDR may involve additional energy requirements (Haspel et al, 1986; Fanciulli et al, 1993). For example, alterations in glucose metabolism are associated with drug resistance in tumour cell lines. One characteristic of malignant cells is an increased rate of oxidative glycolysis and a preferential utilization of oxidative phosphorylation to synthesize ATP (Lyon et al, 1988).

Much of the energy demands are met by glucose transport across the plasma membrane of mammalian cells, mediated by a family of facilitative glucose transporters (GLUT1–4) (Kahn and Flier, 1990; Bell et al, 1993). These exhibit a tissue-specific pattern of expression and distinct kinetic and regulatory properties (Pessin and Bell, 1992). The diversity of the isoforms allows for the regulation of intracellular glucose levels over a range of physiological conditions. GLUT1 is abundantly expressed in erythroid cells; GLUT3 is the primary transporter of glucose in neurons (Bell et al, 1990, 1993). GLUT1 and GLUT3 are considered to be responsible for basal glucose transport in mammalian cells and are localized primarily in the plasma membrane (Asano et al, 1992; Pessin and Bell, 1992). Facilitative glucose transport is an energy-independent saturable process using an alternating conformational model of sugar transport (Walmsley, 1988).

A possible role for the glucose transporter in the modulation of multidrug resistance (MDR) was suggested (Vera et al, 1991). These investigators found that glucose transport inhibitors (GTIs) increased VCR accumulation in *Xenopus* oocytes transfected with GLUT1 mRNA. The effect of GTIs on VCR transport in a MDR cell line also suggested a role for GLUT proteins in VCR transport. We examined the effect of GTIs on vincristine transport in drug-resistant MEL cell lines, which coexpressd GLUT1 and GLUT3 and overexpressed the multidrug resistance-associated protein (MRP) and/or P-glycoprotein (P-gp).

*Received 13 February 1996*
*Revised 30 July 1996*
*Accepted 5 Aug. 1996*

*Correspondence to:* SB Levy, Center for Adaptation Genetics and Drug Resistance, Tufts University School of Medicine, 136 Harrison Ave., Boston, MA 02111, USA

### MATERIALS AND METHODS

### Cell lines

The murine erythroleukaemia (MEL) cell line PC4 and the vincristine (VCR)-selected drug-resistant sublines, named according to the highest drug concentration (ng $ml^{-1}$) into which they were passaged, have been described previously (Slapak et al, 1994). The PC-V40 subline was 42-fold more resistant and PC-V160 was 215-fold more resistant to VCR than the parental cell line. Both sublines demonstrated energy-dependent VCR efflux and overexpressed the multidrug resistance-associated protein (MRP) (Slapak et al, 1996); only PC-V160 cells overexpressed P-glycoprotein (Slapak et al, 1994). Another MEL cell line, C7D (Slapak et al, 1990), and AUXB1 cells (Maher et al, 1992), an auxotrophic CHO cell line (provided by V Ling, Ontario Cancer Institute, Toronto, Canada), were used as controls for GLUT1 and GLUT3 identification in membrane preparations. Cells were grown in Eagle's basal medium (PC4 and C7D) or minimal essential media (AUXB1) supplemented with 10% heat-inactivated fetal calf serum in a 5% carbon dioxide atmosphere (Revco 2000, Ashville, NC, USA).

### Evaluation of steady-state glucose transport

Steady-state glucose transport was measured as a function of the uptake of the glucose analogue 2-deoxy-D-glucose (dGlc) at 50 μM (Asano et al, 1991) in phosphate-buffered saline (PBS) during a 60-min incubation at 37°C. This analogue is not metabolized by mammalian cells beyond the initial phosphorylation step by hexokinase. At this concentration, dGlc presumably represents steady-state glucose levels (Renner et al, 1972). Cells ($1 \times 10^6$ $ml^{-1}$ were preincubated either in the presence of 100 μM phloretin/PBS or PBS alone for 15 min at 37°C. Prior to an additional 60 min, 50 μM dGlc and 5 μCi $ml^{-1}$ (0.096 μM) 2-deoxy-D-[2,6- $^3$H]glucose (52 Ci $mmol^{-1}$; Dupont New England Nuclear, Boston, MA, USA) were added. The reactions were terminated by centrifugation (3 min at $10\,000 \times g$) of 200-μl samples ($2 \times 10^5$ cells) through silicone oil in microfuge tubes previously prepared with 20 μl of formic acid overlaid with 200 μl of silicone oil (D = 1.035–1.045; Nye Lubricants, New Bedford, MA, USA). After freezing the tubes, the tips were severed (containing the frozen formic acid/cell pellet) and the cell-associated radioactivity was determined by scintillation counting (Slapak et al, 1996). The data are expressed as a percentage of cell-associated [$^3$H]dGlc in parental cells after 60 min of incubation from six experiments each performed in duplicate.

### Kinetics of glucose transport

Cells were washed extensively in PBS and resuspended to $1 \times 10^6$ $ml^{-1}$ in PBS at 37°C. 2-deoxy-D-[2,6-$^3$H] glucose ([$^3$H]dGlc)(52 Ci $mmol^{-1}$; Dupont New England Nuclear, Boston, MA, USA) was added (1.6 μCi $ml^{-1}$) to a final concentration of 0.2–2 mM dGlc. Over a 5-min interval, the uptake of [$^3$H]dGlc increased linearly with time for each subline. Therefore, uptake, and not phosphorylation, was the main determinant in cellular dGlc accumulation (Colville et al, 1993). Cell-associated radioactivity was determined after centrifugation of samples ($2 \times 10^5$ cells) through silicone oil (described above) and the cell pellets were quantified for nmol of [$^3$H]dGlc. $K_m$ and $V_{max}$ values were derived from a linear regression of a Lineweaver–Burk graph.

### DNA probes for hybridization

The complete cDNA for rat erythroid/brain hexose transporter, GLUT1, was provided by J Vera (Memorial Sloan Kettering Cancer Research Center, NY, USA). A 1.1-kb probe was generated by digestion with *Nco*I. Rat GLUT2 (prGLUT2), mouse GLUT3 (pmGLUT3-6), rat GLUT4 (prGLUT4) and rat GLUT5 (prGLUT5-4) probes were derived from glucose transporter cDNA clones kindly supplied by Graeme Bell (Howard Hughes Medical Institute, The University of Chicago, USA) (Bell et al, 1993). A β-actin DNA probe (V Stanton, MIT, Cambridge, MA, USA) was used to assess RNA loading. All probes were labelled in-gel with a random primer labelling kit (Boerhinger-Mannheim, Indianapolis, IN, USA) to a specific activity of $1–2 \times 10^9$ c.p.m. $\mu g^{-1}$ DNA.

### RNA extraction and Northern hybridization

RNA was prepared by lysis of $1 \times 10^8$ logarithmically grown cells with guanidine isothiocyanate and then centrifugation through a caesium chloride cushion (Chirgwin et al, 1979). The RNA was analysed by electrophoresis through 1% agarose gels after denaturing by treatment with formaldehyde. The RNA was transferred by blotting to Gene-Screen Plus hybridization membranes (New England Nuclear, Boston, MA, USA). Membranes were prehybridized for 4 h and then hybridized overnight at 42°C in 50% formamide, 5 × SSPE 7.5% dextran sulphate, 1 × Denhardt's solution, 1% sodium dodecyl sulphate (SDS) and 0.2 mg $ml^{-1}$ denatured salmon sperm DNA. The blots were washed to a final stringency of 0.2 × SSPE/0.1% SDS at 56–65°C before exposure to Kodak XAR film at –70°C.

### Membrane isolation

Membrane protein fractions from each subline were prepared as previously described (Vera et al, 1991; Schurmann et al, 1992) with some modifications. Cells ($4 \times 10^8$) were washed twice in PBS and resuspended into 5 ml of lysis buffer (20 mM Tris-Cl, pH 7.4, 1 mM EDTA, 230 mM sucrose containing 20 μg $ml^{-1}$ aprotinin, 20 μg $ml^{-1}$ leupeptin, 50 μg $ml^{-1}$ soybean trypsin inhibitor and 1 mM phenylsulphonylfluoride) and homogenized on ice with a Dounce homogenizer. The homogenates were centrifuged at $1500 \times g$ for 10 min at 4°C to pellet the nuclei. For total membranes, the resultant supernatant was centrifuged at $150\,000 \times g$ for 90 min at 4°C to yield a total membrane fraction, resuspended in lysis buffer and stored at –70°C. For plasma membrane-enriched fraction, the supernatant was centrifuged at $16\,000 \times g$ for 15 min at 4°C. The resulting pellets were resuspended in lysis buffer, layered onto a 1.12 M sucrose cushion (refractive index 1.3902, 38% w/v) and centrifuged for 70 min at $100\,000 \times g$ at 4°C. Plasma membranes were collected from the interface between buffer and sucrose, resuspended in lysis buffer and stored at –70°C. Plasma membrane protein was quantified by assaying the relative activity of alkaline phosphodiesterase I using sodium thymidine 5′-monophosphate, *p*-nitrophenyl ester (Sigma, St Louis, MO, USA) (Draye et al, 1987).

### Analysis of GLUT1 and GLUT3 expression by Western blotting

Plasma membrane protein samples were quantified using lysozyme as a standard, reduced in Laemmli's buffer without boiling (Asano et al, 1989) and resolved by electrophoresis (5 and 1 μg per lane) on an 11% SDS-polyacrylamide minigel (Laemmli, 1970). After electrophoresis, the gels were washed in Towbin's transfer buffer (25 mM Tris, 192 mM glycine) with 20% methanol with 0.01% SDS. Samples were electroblotted overnight (30 V, 100 mA) at 4°C to PVDF membranes (Immobolin P, 0.45 μM pore size; Millipore, Bedford, MA, USA). The membranes were blocked in TBST [20 mM Tris, pH 7.6, 1% bovine serum albumin (BSA) (w/v), 150 mM sodium chloride, 0.05% Tween-20] for 1 h at 25°C.

© Cancer Research Campaign 1997

After three washes in TBST, the membranes were probed with an affinity-purified polyclonal antibody (East Acres Biologicals, Southbridge, MA, USA) to the carboxy terminus of GLUT1 (Haspel et al, 1988) or GLUT3 (Nagamatsu et al, 1992) at a dilution of 1:5000 in TBST for 2 h at 25°C. After three washes in TBST, the immunocomplexes were detected using [$^{125}$I]protein A at 0.01μCi ml$^{-1}$; (117 mCi mg$^{-1}$ ICN) for 1 h at 25°C, washed in TBST and air dried. Autoradiograms were analysed after exposure to Kodak XAR film at –70°C and/or storage phosphor technology (PhosphorImager, Molecular Dynamics, Sunnyvale, CA, USA). Molecular weight standards were electroblotted with samples.

### Quantification of GLUT1 and GLUT3 in the plasma membrane by immunodot blots

Plasma membrane protein samples were processed to Immobolin P membranes pre-equilibrated in Towbin's buffer using a minifold dot blot apparatus (Schleicher & Schuell, Keene, NH, USA). Immobolin P (0.45-μM pore size) was determined by Western blot analysis to retain a maximum of 2.5 μg of MEL plasma membrane protein. Duplicate plasma membrane samples (serial dilutions from 0 to 2.5 μg) were adsorbed by dot blot to PVDF filter membranes and washed in TE (pH 7.4). After Western blotting, the immunocomplexes were detected with a 1:5000 dilution of GLUT1 or GLUT3 antisera as described above. The dot blots were quantified by volume integration of pixels/standard dot blot area using storage phosphor technology (Molecular Dynamics, Sunnyvale, CA, USA) (Johnston et al, 1990) and recorded as pixels μg$^{-1}$ protein for each cell line. The relative level of expression for each isoform (GLUT1 or GLUT3) was normalized to the parental cell line.

### Vincristine steady-state accumulation studies

VCR accumulation was assayed in $10^6$ cells ml$^{-1}$ after 60 min incubation in PBS using 25 nM [G-$^3$H]vincristine sulphate (6.6–8.6 Ci mmol$^{-1}$; Amersham, Arlington Heights, IL, USA) in a 37°C shaking water bath. Steady-state VCR levels were achieved after 60 min of incubation. Cell-associated radioactivity was determined as described above. The data are expressed as a percentage of cell-associated [$^3$H]VCR in parental cells from four separate experiments each performed in triplicate. Surface binding at 4°C was determined to be less than 1% of the total accumulation.

### Effect of glucose transport inhibitors on steady-state vincristine accumulation

Cells (1 × $10^6$ ml$^{-1}$) were preincubated for 15 min at 37°C in phloretin (PHL, 50 μM), cytochalasin B (CB, 6 μM; Sigma, St Louis, MO, USA), cytochalasin E (CE, 6 μM; Sigma) or PBS (control) before the addition of [G-$^3$H]vincristine sulphate (25 nM) for 60 min drug accumulation studies (described above). The data are expressed as a percentage of cell-associated [$^3$H]VCR in parental cells from four separate experiments each performed in triplicate.

The concentration of CB used in these experiments was specific for glucose transport inhibition and minimized other non-glucose transport affinity binding and actin binding (Rampal et al, 1980). CE has a low binding affinity for glucose transport proteins and was used as a control to demonstrate a specific glucose transporter binding by cytochalasin B (Vera et al, 1991). There was no solvent effect on cell viability or VCR uptake with dimethyl sulphoxide (DMSO) (0.08%, CB and CE).

### Effect of phloretin on vincristine efflux

Approximately equimolar intracellular concentrations of VCR were achieved in each subline (PC4-WT, PC-V40 and PC-V160) at 1 × $10^6$ cells ml$^{-1}$ in PBS by adding [G-$^3$H]vincristine sulphate (25 nM, 50 nM and 100 nM respectively during a 60 min incubation in a 37°C shaking water bath. Cells were collected by centrifugation and resuspended to 1 × $10^6$ cells ml$^{-1}$ in drug-free PBS at 37°C. At time zero, phloretin was added to a final concentration of 50 μM. Cells were incubated for 5 min at 37°C; VCR efflux has been demonstrated to be linear for at least 5 min (Slapak et al, 1996). Residual cell-associated radioactivity was determined after centrifugation through silicone oil as described above. There was no solvent effect (0.2% ethanol) on VCR efflux.

### Intracellular ATP levels

Cells were washed directly out of growth media. Employing a luminescence determination (Bioluminescent Somatic Cell Assay Kit, Sigma), duplicate tests with 5 × $10^4$ cells were assayed and light emission was measured by use of a liquid scintillation spectrophotometer (Beckman LS-235, Fullerton, CA, USA) (Stanley and Williams, 1969).

### Method for sequence comparisons

The translation of human MRP (GenBank/EMBL accession no. LO5628) (Cole et al, 1992), mouse MRP (amino acids 601–799; R Deeley, personal communication), mouse GLUT1 (GenBank/EMBL accession no. M23384) (Kaestner et al, 1989) and mouse GLUT3 (GenBank/EMBL accession no. M75135) (Haspel et al, 1988) sequences were analysed using the program Gap from the Sequence Analysis Software Package of the Genetics Computer Group (University Research Park, Madison, WI, USA) (Needleman and Wunsch, 1970). A gap weight of 5.0 and length weight of 0.3 were used in the analyses.

Protein secondary structure predictions of the proposed cytochalasin B binding site in human MRP (amino acids 686–710) (Cole et al, 1992), mouse GLUT1 (amino acids 388–412) (Kaestner et al, 1989) and mouse GLUT3 (amino acids 386–410) (Haspel et al, 1988) were performed using the MacVector program. Hydropathy plots were graphed using the Kyte–Doolittle scale and a window of seven amino acids (Kyte and Doolittle, 1982).

### Statistical analyses

Paired *t*-tests were used to evaluate statistical significance in drug accumulation studies, drug efflux studies, hexose transport studies and PhosphorImager calculations. Linear regression of Lineweaver–Burk plots was used in the analysis of hexose transport kinetic studies ($K_m$ and $V_{max}$). All statistics were calculated with software program Statview (Brainpower Inc., Calabasas, CA, USA) and DeltaGraph 1.5 (Delta Point Inc., Monterey, CA, USA).

### RESULTS

### Steady-state glucose accumulation: effect of phloretin

The steady-state accumulation of glucose, as judged by accumulation of [$^3$H]dGlc, increased in cells with progressive VCR resistance. The PC-V40 subline accumulated 1.5-fold more and the PC-V160 subline accumulated twofold more [$^3$H]dGlc than the parental cell

© Cancer Research Campaign 1997

**Figure 1** Effect of phloretin on 2-deoxy-D-glucose (dGlc) accumulation in parental and drug-resistant cell lines. Cells ($1 \times 10^6$ ml$^{-1}$) were preincubated either in the presence of 100 μM phloretin/PBS or PBS alone for 15 min at 37°C. A total of 50 μM dGlc and 0.096 μM [$^3$H]dGlc were added and the cells were incubated for an additional 60 min. The reactions were terminated by centrifugation through silicone oil and the uptake determined. The data are expressed as a percentage of cell-associated [$^3$H]dGlc in parental cells after 60 min of incubation. The average uptakes (± s.e.) from six experiments each performed in duplicate are shown. ■, control; ■, phloretin (100 μM)

**Figure 2** Kinetics of glucose transport in PC4 cell lines. A Lineweaver–Burk plot of transport data is shown. Cells were washed once in PBS and resuspended to $1 \times 10^6$ cells ml$^{-1}$ in PBS. dGlc was added to a final concentration of 0.2–5.0 mM and 0.031 μM [$^3$H]dGlc. Duplicate samples were analysed for each dGlc concentration in three separate experiments at 37°C

**Table 1** Glucose transport kinetics

| | $K_m$[a] (mM) | $V_{max}$[a] (pmol dGlc min$^{-1}$ per $2 \times 10^5$ cells) | Relative index of glucose transport[b] |
|---|---|---|---|
| PC4-WT | 1.49 ± .24 | 6.6 ± 4.9 | 1 |
| PC-V40 | 1.35 ± .46 | 12.5 ± 1.8 | 1.9 |
| PC-V160 | 1.45 ± .26 | 16.8 ± 3.1 | 2.5 |

[a]Values are derived from a linear regression of a Lineweaver–Burk plot (Figure 2). [b]The $V_{max}$ values were normalized to the parental cell line.

line (Figure 1). The transport of glucose was inhibited approximately 85–90% ($P \leq 0.05$) by the competitive glucose transport inhibitor, phloretin (100 μM) (Figure 1). As a competitive inhibitor of glucose transport, the effect of phloretin differentiated facilitative glucose transport from simple hexose diffusion (Krupka and Deves, 1980; Wheeler and Hinkle, 1985). These findings suggested that dGlc, and therefore glucose, uptake occurred primarily via a facilitative glucose transporter(s) in the PC4 sublines.

### Kinetic analysis of glucose transport

The kinetics of glucose transport were evaluated in the sublines (Table 1 and Figure 2). Increases in $V_{max}$ were observed: 1.9-fold increase in PC-V40 and a 2.5-fold increase in the PC-V160 cell line. The high correlation coefficients ($r$) calculated from the linear regression of the glucose transport studies suggested unidirectional glucose transport into the cells (Asano et al, 1989; Colville et. al, 1993). The $K_m$ values (determined by [$^3$H]dGlc uptake) were within the reported range for mammalian erythroid glucose transporter GLUT1 (Asano et al, 1991, 1992) and for the mammalian brain glucose transporter, GLUT3 (Asano et al, 1992; Bell et al, 1993).

### Analysis of GLUT expression by Northern hybridization

Total RNA from each subline was examined by Northern analysis with rodent-specific cDNA probes for the GLUT1–5 transcripts. Under high-stringency hybridization conditions, expression of both GLUT1 and GLUT3 was detected in the parental and VCR-selected sublines (Figure 3A). Expression of GLUT2, GLUT4 and GLUT5 was not detected even with prolonged film exposure. The mRNA transcripts of GLUT1 and GLUT3 were approximately 2.8 kb. The GLUT3 hybridized to a smaller transcript (2.8 kb) in these erythroid cells than the size (4.0 kb) reported previously for neuronal cells (Maher et al, 1992); however, a 2.7-kb transcript has been noted in non-neuronal tissues (Yano et al, 1991). The level of total GLUT mRNA in the parental cell line was reproducible at a higher level than in the more resistant cell lines. Reprobing with the β-actin probe demonstrated near equal RNA sample loading.

### Analysis of GLUT1 and GLUT3 protein expression

The level of GLUT protein found in total cell membranes mirrored the pattern of GLUT1 and GLUT3 RNA expression: somewhat less protein was seen in the membranes from the PC-V40 and PC-V160 sublines (data not shown). However, this pattern was not seen in the Western blot analyses of the plasma membrane protein-enriched preparations (Figure 3B). Here, instead, the level of expression of broadly migrating polypeptides of $M_r$ 47–51 (GLUT1) and $M_r$ 48–52 (GLUT3) increased with VCR resistance.

Immunodot blots probed with GLUT1 or GLUT3 antisera provided additional quantitative analysis of the plasma membrane GLUT protein content among these sublines. GLUT1 protein

 © Cancer Research Campaign 1997

C

| WT | V40 | V160 | |
|---|---|---|---|
| 1.0 | 1.4 ± 0.20 | 3.3 ± 0.53 | GLUT1 |
| 1.0 | 1.1 ± 0.04 | 3.0 ± 0.04 | GLUT3 |

**Figure 3** Expression of GLUT1 and GLUT3 in parental and drug-resistant cell lines. (**A**) Northern blots. RNA was isolated from parental PC4 (WT) and PC-V40 and PC-V160 sublines. The RNA was resolved by electrophoresis (20 µg per lane), transferred to a nylon membrane, then sequentially hybridized with $^{32}$P-labelled cDNA probes for rat GLUT1, rat GLUT2, mouse GLUT3, rat GLUT4 and rat GLUT5. A hybridization signal (corresponding to a 2.8-kb transcript) was detected only with the GLUT1 and GLUT3 probe. Rehybridization with cDNA for β-actin confirmed near-equal loading of lanes. (**B**) Western blots. Plasma membrane protein samples (5 and 1 µg per lane) from the parental, PC-V40 and PC-V160 cells were resolved by electrophoresis on an 11% SDS-polyacrylamide gel. Western blotting was performed with a GLUT1 or GLUT3 antisera at a dilution of 1:5000, and immunocomplexes were detected with [$^{125}$I]protein A and autoradiography. Immunoblots detected broadly migrating polypeptides of $M_r$ 47–51 (GLUT1) and $M_r$ 48–52 (GLUT3). (**C**) Plasma membrane GLUT1 and GLUT3 protein expression by immunodot blot. Plasma membrane samples were adsorbed by dot blot to Immobolin P membranes. After transfer, Western blotting was performed using GLUT1 or GLUT3 antisera. Immunocomplexes were detected with [$^{125}$I]protein A and quantified by volume integration of pixels per standard dot blot area using storage phosphor technology (Johnston et al, 1990) and recorded as pixels µg$^{-1}$ protein for each cell line. Each value in the table represents the mean and standard error from five protein dilutions calculated as the ratio of subline to parental pixel values expression was 40% higher in the plasma membranes from the PC-V40 subline and threefold higher in those from the PC-V160 subline (Figure 3C). There was little change in the level of GLUT3 protein expression in the PC-V40 cells, but a threefold increase in the PC-V160 cells (Figure 3C). These findings paralleled the relative increase in $V_{max}$ (Table 1) for glucose uptake in these sublines. The equal activity of alkaline phosphodiesterase I, a plasma membrane-associated enzyme, confirmed equal amounts of the protein samples being tested.

AUXB1, a Chinese hamster ovarian cell line, a previously reported (Maher et al, 1992) GLUT1-expressing, GLUT3-non-expressing cell line provided positive and negative controls for the antisera. Another separately derived MEL cell line, C7D (Slapak et al, 1990), also demonstrated coexpression of GLUT1 and GLUT3 with protein sizes equal to those seen in the PC4-WT cell line (data not shown).

### Effect of glucose transport inhibitors on vincristine accumulation

The effect of glucose transport inhibitors (GTIs) on the 60-min VCR accumulation in both non-P-gp- and P-gp-expressing MEL cell lines was evaluated (Figure 4) in PBS without glucose to minimize the interference of glucose with the binding by these inhibitors.

Compared with the PC4-WT cells, the VCR accumulation in PBS alone was reduced by 41% ($P \leq 0.05$) in the PC-V40 subline and 79% ($P < 0.05$) in PC-V160 subline (Figure 4). These findings were similar to the previous studies in the presence of glucose (Slapak et al, 1996).

Pretreatment and incubation of the cell lines in 6 µM cytochalasin B (CB), a competitive glucose transport inhibitor (Deves and Krupka, 1978; Wheeler and Hinkle, 1985; Holman and Rees, 1987; Garcia et al, 1992), increased the steady-state VCR accumulation in the PC-V40 cell line to the level of PC4-WT cells ($P \leq 0.05$) (Figure 4). In contrast, 6 µM cytochalasin E (CE), used to assess any effects of cytochalasin independent of the glucose transport inhibition, did not cause a statistically significant

**Figure 4** Effect of cytochalasin B and E on the accumulation of VCR in PC4 cell lines. PC4 cell lines (1 × 10$^6$ cells ml$^{-1}$) were preincubated in cytochalasin B/PBS (6µM), cytochalasin E/PBS (6 µM) or control/PBS for 15 min at 37°C. [$^3$H]VCR (25 nM) was added and cells were incubated for an additional 60 min at 37°C. [$^3$H]VCR uptake was determined as described in Figure 1. Shown are the mean (± s.e.) of 3–4 separate experiments performed in triplicate. *Significantly ($P \leq 0.05$) increased intracellular [$^3$H]VCR compared with its own subline control

**Table 2** Effect of phloretin on vincristine efflux in PC4 cell lines

| | Loss of intracellular vincristine[a] (%) | |
|---|---|---|
| | Control | Phloretin |
| PC4-WT | 15 | 7 |
| PC-V40 | 32 | 15[b] |
| PC-V160 | 46 | 41 |

[a]Cells were incubated in [$^3$H]VCR for 60 min in growth media without serum at 37°C at a concentration of [$^3$H]VCR to obtain equimolar drug concentrations: PC4-WT, 25 nM; PC-V40, 50 nM; PC-V160, 100 nM. Cells were washed out of drug and resuspended in PBS with 50 μM phloretin. After 5 min of incubation at 37°C, the reaction was terminated by centrifugation through silicone oil and cell-associated radioactivity was determined as described in Figure 1. The % loss of initially accumulated vincristine by 5 min is reported. [b]Represents significantly ($P \leq 0.05$) increased intracellular [$^3$H]VCR accumulation in PC-V40 with phloretin.

**Figure 5** (**A**) Alignment of residues present in mouse GLUT1 and mouse GLUT3 in the proximity of the proposed cytochalasin B-binding site to mouse MRP. The amino acids are presented as aligned by the GCG program Gap (Needleman and Wunsch, 1970). Identical pairings are represented by lines (|) and variable values of similarity are represented by dots (: or.). The peptide sequences in mouse and human (not shown) MRP represent amino acids 686–710 and correspond to a region between the Walker A and B motifs within the N-terminal ATP-binding domain of the human MRP protein (Cole et al, 1992; R Deeley, personal communication). The amino acid sequences Trp388–Trp412 in mouse GLUT1 and Trp386–Trp410 in mouse GLUT3 are located within the transmembrane 10–11 segments. These regions contain the putative CB-binding sites within the glucose transporters (Holman and Rees, 1987; Garcia et al, 1992). The motif AxLxxxxxxxxxxxxxG (x is any amino acid) was found within the mouse MRP (R Deeley, personal communication), human MRP (Cole et al, 1992), mouse GLUT1 (Kaestner et al, 1989), mouse GLUT3 (Nagamatsu et al, 1992) and mouse GLUT4 (Kaestner et al, 1989) proteins. (**B**) Hydropathy plots of the proposed cytochalasin B-binding site. The peptide sequences from mouse MRP (686–710), mouse GLUT1 (388–412) and mouse GLUT3 (386–410) were graphed using the MacVector program with a Kyte–Doolittle scale and a window of seven amino acids (Kyte and Doolittle, 1982)

reversal of the [$^3$H]VCR accumulation deficit in the PC-V40 cell line (Figure 4). Neither CB nor CE statistically increased VCR accumulation in PC4-WT or PC-V160 cells. The absence of an effect by CE suggested that CB's activity was linked to its affinity for the glucose transporter (Rampal et al, 1980; Vera et al, 1991).

### Effect of phloretin on vincristine efflux

Increased vincristine efflux has been demonstrated in PC-V40 and PC-V160 cells; efflux was linear for the first 5 min (Slapak et al, 1996). VCR efflux was evaluated in the presence of phloretin (PHL). PHL diminished the VCR efflux rate in the PC-V40 subline to nearly that of the PC4-WT subline control ($P \leq 0.05$) (Table 2). This inhibitor had no significant effect on VCR efflux in PC4-WT or PC-V160 cells.

### Analysis of intracellular ATP content

ATP levels in cells grown in media was $4.1 \times 10^{-15}$ mol per cell ± 0.90 (s.e.). There was no change in ATP content in the cell lines after a total of 75 min incubation in either CB (6 μM)/PBS or CE (6 μM)/PBS during steady-state VCR accumulation studies.

### Sequence comparisons of the MRP, GLUT1 and GLUT3 proteins

The gene sequences of human MRP (Cole et al, 1992), mouse GLUT1 (Kaestner et al, 1989) and mouse GLUT3 (Haspel et al, 1988) were aligned using the GCG program, Gap. The overall deduced proteins' percentage amino acid identities/similarities were as follows: human MRP/mouse GLUT1, 12/35 (5 gaps); human MRP/mouse GLUT3, 15/43 (13 gaps); and mouse GLUT1/mouse GLUT3, 64/80 (0 gaps). The murine MRP sequence has not yet been reported, but we have learned that the deduced amino acid sequence of murine and human MRP showed a greater than 90% identity (R Deeley, personal communication).

Gap analyses (Figure 5A) were also performed to determine the possible presence of a CB-binding site in the MRP protein. Amino acids Trp-388–Trp-412 in mouse GLUT1 and Trp-386–Trp-410 in mouse GLUT3 are located within the putative CB-binding site within the transmembrane 10–11 segments (Holman and Rees, 1987; Garcia et al, 1992). These regions of GLUT1 and GLUT3 were aligned with the Gap program to the human MRP protein (Cole et al, 1992) revealing a corresponding region between the Walker A and B motifs within the putative N-terminal ATP-binding domain (amino acids 686–710). From the deduced amino acid sequence of this region of the mouse MRP (received from R Deeley), a 92–96% identity/similarity with human MRP was found. The percentage amino acid identities/similarities from these comparisons showed mouse MRP/mouse GLUT1, 12/44; mouse MRP/mouse GLUT3, 12/44; and mouse GLUT1/mouse GLUT3, 88/92 (0 gaps) (Figure 5A).

Other related gene sequences (Szczypka et al, 1994) were aligned to the putative CB-binding domains on GLUT1 and GLUT3 using Gap: (1) mouse CFTR; (2) mouse P-glycoprotein (*mdr1* but not *mdr3*); (4) *Leishmania* P-gpA; (5) *S. cerevisiae* YCF1 (yeast cadmium factor); and (6) human P-gp (*MDR1*). A similar region of homology within the highly conserved nucleotide-binding regions to the GLUT1 or GLUT3 CB-binding sites was not found in any but human P-gp. Here a region of homology to the GLUT3 CB-binding site was noted within the N-terminal ATP-binding fold; however, it did not correspond to the same numerical residues nor did it have the 'conserved motif' AxLxxxxxxxxxxAxxG. The hydropathy plots of mouse GLUT1, GLUT3 and MRP in this region demonstrated a similar pattern, including two hydrophobic regions with an intervening short hydrophilic region (Figure 5B).

© Cancer Research Campaign 1997

### DISCUSSION

In multidrug-resistant murine erythroleukaemia cells, glucose transport activity increased with increased drug resistance and was associated with coexpression of two GLUT proteins, GLUT1 and GLUT3. Only GLUT1 is normally expressed in erythroid cells. Mouse GLUT1 has a widespread tissue distribution (Kahn and Flier, 1990; Pessin and Bell, 1992; Bell et al, 1993) and was expected in this murine erythroleukaemia cell line. Not expected was the presence of the mouse GLUT3 isoform. These findings indicated a less restricted tissue distribution for the GLUT3 isoform than has previously been reported (Gould et al, 1992) and represented a novel murine (non-neuronal) GLUT3 tissue expression.

The GLUT1 and GLUT3 isoforms are primarily translocated and expressed at the plasma membrane and regulate basal glucose metabolism (Asano et al, 1992; Pessin and Bell, 1992; Schurmann et al, 1992). The levels of GLUT1 and GLUT3 protein in the total membrane of the cell lines correlated with the relative GLUT1 and GLUT3 mRNA expression (Figure 4), but these levels did not correlate with glucose transport. The PC-V40 expressed both a reduced level of mRNA and total protein, but showed increased glucose transport. This discrepancy was clarified by Western blot analysis of the plasma membrane. There, the level of GLUT1 and GLUT3 proteins correspondingly increased with the level of glucose transport verifying the important location of the glucose transporters.

The discordance between mRNA and protein levels and glucose transport in the PC-V40 cell line may be caused by alterations in metabolic state, in the stability or translatability of the mRNA or the stability of the protein (Yamada et al, 1983; Haspel et al, 1986) and could reflect an adaptive response to an increased energy requirement associated with expression of *mrp* (PC-V40 subline) or *mdr3* (PC-V160 subline). In multidrug-resistant MEL cells, the increased levels of glucose transport protein in the plasma membrane paralleled the increased rates of vincristine efflux and VCR resistance.

The possible participation of GLUT1 in drug resistance was previously suggested (Vera et al, 1991). CB and PHL, two inhibitors of glucose transport, could overcome the reduced Vinca alkaloid, vinblastine, accumulation conferred by the expression of rat GLUT1 in *Xenopus* oocytes. In their drug-selected MDR cell line expressing *mdr1* (the level of MRP was not evaluated in these cell lines), CB was able to inhibit the outward transport of vinblastine. However, in transfected oocytes expressing P-gp, this GTI effect was not demonstrated.

In vincristine-selected PC4 cell lines, murine MRP was expressed primarily in the plasma membrane (Slapak et al, 1996). The MRP-overexpressing cell line, PC-V40, was capable of an energy-dependent outward efflux of VCR, independent of P-glycoprotein. The cell line PC-V160 demonstrated a greater rate of VCR efflux with coexpression of MRP and P-gp. However, compared with the PC-V40 cell line, PC-V160's level of MRP was significantly less. A possible association between MRP and/or P-gp function and plasma membrane-associated GLUT protein expression was evaluated by the effect of GTIs on VCR transport in the cell lines, all of which coexpressed GLUT1 and GLUT3. CB reversed the deceased VCR accumulation in PC-V40, the cell line that overexpressed MRP. No such CB effect was seen in the PC-V160 cell line, which overexpressed both MRP and P-gp. CE at the same concentration did not affect the accumulation of VCR in any of the cell lines. Since CE has an approximately 20-fold greater incorporation into actin-binding sites than CB, our results indicate that neither CB nor CE at these concentrations affected actin and presumably the intracellular cytoskeletal function. Another inhibitor, PHL, reduced the rate of VCR efflux in PC-V40 to the level of the parental cell line. In contrast, PHL did not affect VCR efflux in the PC-V160 cell line or parental cell line. More recent studies have shown that indomethacin, a specific inhibitor of MRP, affected VCR resistance in PC-V40, but not VCR resistance in PC-V160 (Draper et al, submitted). Taken together these data suggest that VCR efflux was primarily associated with MRP overexpression in the PC-V40 cell line and with P-glycoprotein overexpression in the PC-V160 cell line.

CB has been demonstrated to inhibit glucose transport competitively in erythrocytes (Deves and Krupka, 1978). It may bind at or near the inward-facing glucose-binding site of GLUT1 in the region of Trp-388 to Trp-412 within the transmembrane segment 10–11 (Garcia et al, 1992). The amino acid sequences representing the putative CB-binding domain in the GLUT1 and GLUT3 proteins were compared with the protein sequence of the MRP protein (Cole et al, 1992). A single polypeptide sequence located between the Walker A and B motifs within the putative N-terminal ATP-binding cassette domain of human MRP showed similarity to the CB-binding site located within the transmembrane 10–11 segments of the GLUT1 and GLUT3 proteins (Figure 5 legend). The similar hydropathy patterns in these regions suggested possible structural similarities in this sequence among these proteins (Figure 5). The binding domain of phloretin on glucose transport proteins is not well characterized (Krupka and Deves, 1980; Wheeler and Hinkle, 1985).

The results suggest that CB and PHL block a putative VCR efflux protein, e.g. MRP, or act directly on one or both GLUT proteins, which themselves are somehow associated with drug efflux. It is possible that glucose transporters could in some manner modulate MRP's ability to transport VCR out of the cell. That energy reserves (ATP) were not depleted during the transport inhibition studies indicated that intracellular energy depletion was not responsible for the GTI's effect on VCR efflux in PC-V40 cells. Understanding the mode of action of GTIs on MRP-mediated resistance may help us understand MRP function better, as well as the possible role of GLUT proteins in MRP activity.

### ABBREVIATIONS

MRP, multidrug resistance-associated protein; P-gp, P-glycoprotein; GLUT1, type 1 isoform of the facilitative glucose transporter; GLUT3, type 3 isoform of the facilitative glucose transporter; VCR, vincristine; MDR, multidrug resistance; dGlc, 2-deoxy-D-glucose; PBS, phosphate-buffered saline; PHL, phloretin; CB, cytochalasin B; CE, cytochalasin E.

### ACKNOWLEDGEMENTS

We thank Roger Deeley for sharing the relevant mouse MRP sequence with us, and Graeme Bell, Juan Vera and V Stanton for valuable DNA probes. The authors acknowledge helpful discussions with Michael Draper and Laura McMurry. This investigation was supported in part by USPHS Awards T32-CA09429 (RLM), CA-593451 (SBL), Clinical Investigator Award CA-01613 (CAS) and the Arnold D Imperatore Research Scholarship of the National Leukemia Research Association (SBL).

© Cancer Research Campaign 1997

### REFERENCES

Asano T, Shibasaki Y, Ohno S, Taira H, Lin J-L, Kasuga M, Kanazawa Y, Akanuma Y, Takaku F and Oka Y (1989) Rabbit brain glucose transporter responds to insulin when expressed in insulin-sensitive Chinese hamster ovary cells. *J Biol Chem* **264**: 3416–3420

Asano T, Shibasaki Y, Lin J-L, Tsukuda K, Katagiri H, Ishihara H, Yazaki Y and Oka Y (1991) Expression of the GLUT1 glucose transporter increases thymidine uptake in Chinese hamster ovary cells at low glucose concentrations. *Cancer Res* **51**: 4450–4454

Asano T, Katagiri H, Takata K, Tsukuda K, Lin J-L, Ishihara H, Inukai K, Hirano H, Yazaki Y and Oka Y (1992) Characterization of GLUT3 protein expressed in Chinese hamster ovary cells. *Biochem J* **288**: 189–193

Bell GI, Kayano T, Buse JB, Burant CF, Takeda J, Lin D, Fukumoto H and Seino S (1990) Molecular biology of mammalian glucose transporters. *Diabetes Care* **13**: 198–208

Bell GI, Burant CF, Takeda J and Gould GW (1993) Structure and function of mammalian facilitative sugar transporters. *J Biol Chem* **268**: 19161–19164

Chirgwin JM, Przbyla AE, Macdonald RJ and Rutter WJ (1979) Isolation of biologically active ribonucleic acid from sources enriched in ribonuclease. *Biochemistry* **18**: 5294–5299

Cole SPC, Bhardwaj G, Gerlach JH, Mackie JE, Grant CE, Almquist KC, Stewart AJ, Kurz EU, Duncan AMV and Deeley RG (1992) Overexpression of a transporter gene in a multidrug-resistant human lung cancer cell line. *Science* **258**: 1650–1654

Cole SPC, Sparks KE, Fraser K, Loe DW, Grant CE, Wilson GM and Deeley RG (1994) Pharmacological characterization of multidrug resistant MRP-transfected human tumor cells. *Cancer Res* **54**: 5902–5910

Colville CA, Seatter MJ, Jess TJ, Gould GW and Thomas HM (1993) Kinetic analysis of the liver-type (GLUT2) and brain-type (GLUT3) glucose transporters in *Xenopus* oocytes: substrate specificities and effects of transport inhibitors. *Biochem J* **290**: 701–706

Deves R and Krupka RM (1978) Cytochalasin B and the kinetics of inhibition of biological transport. *Biochim Biophys Acta* **510**: 339–348

Draper MP, Martell RL and Levy SB (1997) Indomethacin-mediated reversal of multidrug resistance and drug efflux in human and murine cell lines overexpressing MRP, but not P-glycoprotein. *Br J Cancer* (in press)

Draye J-P, Courtoy PJ, Quintart J and Baudhuin P (1987) Relations between plasma membrane and lysosomal membrane: quantitative evaluation of plasma membrane marker enzymes in the lysosomes. *Eur J Biochem* **170**: 405–411

Fanciulli M, Bruno T, Castiglione S, Del Carlo C, Paggi MG and Floridi A (1993) Glucose metabolism in adriamycin-sensitive and -resistant Lo Vo human colon carcinoma cells. *Oncology Res* **5**: 357–62

Garcia JC, Strube M, Leingang K, Keller K and Mueckler MM (1992) Amino acid substitutions at tryptophan 388 and tryptophan 412 of the HepG2 (Glut 1) glucose transporter inhibit transport activity and targeting to the plasma membrane in *Xenopus* oocytes. *J Biol Chem* **267**: 7770–7776

Gottesman MM and Pastan I (1993) Biochemistry of multidrug resistance mediated by the multidrug transporter. *Annu Rev Biochem* **62**: 385–427

Gottesman MM (1993) How cancer cells evade chemotherapy: Sixteenth Richard and Hinda Rosenthal Foundation Award lecture. *Cancer Res* **53**: 747–754

Gould GW, Brant AM, Kahn BB, Shepherd PR, Mccoid SC and Gibbs EM (1992) Expression of the brain-type glucose transporter is restricted to brain and neuronal cells in mice. *Diabetologia* **35**: 304–309

Haspel HC, Wilk EW, Birnbaum MJ, Cushman SW and Rosen OM (1986) Glucose deprivation and hexose transporter polypeptides of murine fibroblasts. *J Biol Chem* **261**: 6778–6789

Haspel HC, Rosenfeld MG and Rosen OM (1988) Characterization of antisera to a synthetic carboxy-terminal peptide of the glucose transporter protein. *J Biol Chem* **263**: 398–403

Holman GD and Rees WD (1987) Photolabelling of the hexose transporter at external and internal sites: fragmentation patterns and evidence for a conformational change. *Biochim Biophys Acta* **897**: 395–405

Johnston RF, Pickett SC and Barker DL (1990) Autoradiography using storage phosphor technology. *Electrophoresis* **11**: 355–360

Juranka PF, Zastawny RL and Ling V (1989) P-glycoprotein: multidrug resistance and a superfamily of membrane-associated transport proteins. *Faseb J* **3**: 2583–2592

Kaestner KH, Christy RJ, Mclenithan JC, Braiterman LT, Cornelius P, Pekala PH and Lane MD (1989) Sequence, tissue distribution, and differential expression of mRNA for a putative insulin-responsive glucose transporter in mouse 3T3-L1 adipocytes. *Proc Natl Acad Sci USA* **86**: 3150–3154

Kahn BB and Flier JS (1990) Regulation of glucose-transporter gene expression *in vitro* and *in vivo*. *Diabetes Care* **13**: 548–564

Krupka RM and Deves R (1980) Asymmetric binding of steroids to internal and external sites in the glucose carrier erythrocytes. *Biochim Biophys Acta* **598**: 134–144

Kyte G and Doolittle RF (1982) A simple method for displaying the hydropathic character of a protein. *J Mol Biol* **157**: 105–132

Laemmli UK (1970) Clevage of structural proteins during the assembly of the head of bacteriophage T4. *Nature* **227**: 680–685

Lyon RC, Cohen JS, Faustino PJ, Megnin F and Myers CE (1988) Glucose metabolism in drug-sensitive and drug-resistant human breast cancer cells monitored by magnetic resonance spectroscopy. *Cancer Res* **48**: 870–877

Maher F, Vannucci S, Takeda J and Simpson IA (1992) Expression of mouse-GLUT3 and human-GLUT3 glucose transporter proteins in brain. *Biochem Biophys Res Commun* **182**: 703–711

Nagamatsu S, Kornhauser JM, Burant CF, Seino S, Mayo KE and Bell GI (1992) Glucose transporter expression in brain: cDNA sequence of mouse GLUT3, the brain facilitative glucose transporter isoform, and identification of sites of expression by *in situ* hybridization. *J Biol Chem* **267**: 467–472

Needleman SB and Wunsch CD (1970) A general method applicable to the search for similarities in the amino acid sequence of two proteins. *J Mol Biol* **48**: 443–453

Pessin JE and Bell GI (1992) Mammalian facilitative glucose transporter family: structure and molecular regulation. *Annu Rev Physiol* **54**: 911–930

Rampal AL, Pinkofsky HB and Jung CY (1980) Structure of cytochalasins and cytochalasin B binding sites in human erythrocytes membranes. *Biochemisitry* **19**: 679–683

Renner ED, Plagemann PGW and Bernlohr RW (1972) Permeation of glucose by simple and facilitated diffusion by Novikoff rat hepatoma cells in suspension culture and its relationship to glucose metabolism. *J Biol Chem* **247**: 5765–5776

Schurmann A, Monden I, Joost HG and Keller K (1992) Subcellular distribution and activity of glucose transporter isoforms GLUT1 and GLUT4 transiently expressed in COS-7 cells. *Biochim Biophys Acta* **1131**: 245–252

Slapak CA, Daniel JC and Levy SB (1990) Sequential emergence of distinct resistance phenotypes in murine erythroleukemia cells under adriamycin selection: decreased anthracycline uptake precedes increased P-glycoprotein expression. *Cancer Res* **50**: 7895–7901

Slapak CA, Fracasso PM, Martell RL, Toppmeyer DL, Lecerf JM and Levy SB (1994) Overexpression of the multidrug resistance-associated protein (MRP) gene in vincristine but not doxorubicin-selected multidrug-resistant murine erythroleukemia cells. *Cancer Res* **54**: 5607–5613

Slapak CA, Martell RL, Terashima M and Levy SB (1996) Increased efflux of vincristine, but not of daunorubicin, associated with the murine multidrug resistance-associated protein (MRP). *Biochem Pharmacol* **52**: 1569–1576

Stanley PE and Williams SG (1969) Use of the liquid scintillation spectrometer for determining adenosine triphosphate by the luciferase enzyme. *Anal Biochem* **29**: 381–392

Szczypka MS, Wemmie JA, Moye-Rowley WS and Thiele DJ (1994) A yeast metal resistance protein similar to human cystic fibrosis transmembrane conductance regulator (CFTR) and multidrug resistance-associated protein. *J Biol Chem* **269**: 22853–22587

Tew KD (1994) Glutathione-associated enzymes in anticancer drug resistance. *Cancer Res* **54**: 4313–4320

Vera JC, Castillo GR and Rosen OM (1991) A possible role for a mammalian facilitative hexose transporter in the development of resistance to drugs. *Mol Cell Biol* **11**: 3407–3418

Walmsley AR (1988) The dynamics of the glucose transporter. *Trends Biochem Sci* **13**: 226–231

Wheeler TJ and Hinkle PC (1985) The glucose transporter of mammalian cells. *Annu Rev Physiol* **47**: 503–517

Yamada K, Tillotson LG and Isselbacher KJ (1983) Regulation of hexose carriers in chicken embryo fibroblasts: effect of glucose starvation and role of protein synthesis. *J Biol Chem* **258**: 9786–9792

Yano H, Seino Y, Inagaki N, Hinokio Y, Yamamoto T, Yasuda K, Masuda K, Someya Y and Imura H (1991) Tissue distribution and species difference of the brain type glucose transporter (GLUT3). *Biochem Biophys Res Commun* **174**: 470–477

© Cancer Research Campaign 1997